# Independent effects of *ADH1B* and *ALDH2* common dysfunctional variants on gout risk

**DOI:** 10.1038/s41598-017-02528-z

**Published:** 2017-05-31

**Authors:** Masayuki Sakiyama, Hirotaka Matsuo, Airi Akashi, Seiko Shimizu, Toshihide Higashino, Makoto Kawaguchi, Akiyoshi Nakayama, Mariko Naito, Sayo Kawai, Hiroshi Nakashima, Yutaka Sakurai, Kimiyoshi Ichida, Toru Shimizu, Hiroshi Ooyama, Nariyoshi Shinomiya

**Affiliations:** 1 Department of Integrative Physiology and Bio-Nano Medicine, National Defense Medical College, 3-2 Namiki, Tokorozawa, Saitama 359-8513 Japan; 2 Department of Dermatology, National Defense Medical College, 3-2 Namiki, Tokorozawa, Saitama 359-8513 Japan; 3 Department of Preventive Medicine, Nagoya University Graduate School of Medicine, 65 Tsurumai-cho, Showa-ku, Nagoya, Aichi 466-8550 Japan; 4 Department of Preventive Medicine and Public Health, National Defense Medical College, 3-2 Namiki, Tokorozawa, Saitama 359-8513 Japan; 5 Department of Pathophysiology, Tokyo University of Pharmacy and Life Sciences, 1432-1 Horinouchi, Hachiouji, Tokyo 192-0392 Japan; 6 Kyoto Industrial Health Association, 67 Kitatsuboi-cho, Nishinokyo, Nakagyo-ku, Kyoto 604-8472 Japan; 7 Ryougoku East Gate Clinic, 3-21-1 Ryougoku, Sumida-ku, Tokyo 130-0026 Japan

## Abstract

Gout is caused by hyperuricemia, with alcohol consumption being an established risk factor. Alcohol dehydrogenase (ADH) and aldehyde dehydrogenase (ALDH) are crucial enzymes for alcohol metabolism. We recently performed a genome-wide association study of gout and a subsequent fine-mapping study which identified rs671 of *ALDH2* as a gout locus. However, the association between gout and common variants of *ADH1B* has hitherto remained unreported, prompting us to investigate the association between gout and common dysfunctional variants of *ADH1B* (rs1229984) and *ALDH2* (rs671). We used 1,048 clinically defined gout cases and 1,334 controls of Japanese male. The “His carrier” (His/His or His/Arg) of rs1229984 (His48Arg) of *ADH1B* significantly increased gout risk (*P* = 4.3 × 10^−4^, odds ratio = 1.76), as did the “non-Lys carrier (Glu/Glu)” of rs671 (Glu504Lys) of *ALDH2*. Furthermore, common variants of *ADH1B* and *ALDH2* are independently associated with gout. Our findings likewise suggest that genotyping these variants can be useful for the evaluation of gout risk.

## Introduction

**Figure 1:**
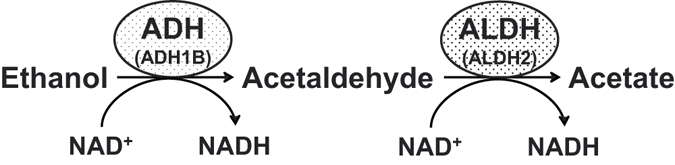
Ethanol oxidization by ADH and ALDH. Ethanol is oxidized to acetaldehyde by alcohol dehydrogenase (ADH), and acetaldehyde is further metabolized to acetate by aldehyde dehydrogenase (ALDH). These oxidization activities largely depend on ADH1B and ALDH2, respectively. The enzymatic activities of ADH1B and ALDH2 depend on common dysfunctional variants, rs1229984 (His48Arg) and rs671 (Glu504Lys) in East Asians, respectively. The A/A (His/His) or A/G (His/Arg) genotype of rs1229984 has been reported to produce 40-fold faster ethanol oxidation by ADH1B than the G/G (Arg/Arg) genotype. Individuals with heterozygotes (Lys/Glu) of rs671 have only 6.25% of the enzyme activity of those with normal ALDH2 (Glu/Glu), and those with homozygotes (Lys/Lys) show almost no activity. Therefore, the Lys carrier of *ALDH2* metabolizes acetaldehyde more slowly than the non-Lys carrier, resulting in acetaldehyde accumulation.

Gout is an increasingly common disease resulting from hyperuricemia, which causes acute arthritis. Several genes have been reported to be associated with gout^1–5^. Some urate transporter genes, such as *ABCG2* ^6–8^, *SLC2A9* ^3, 4^, *SLC17A1* ^3, 9^ and *SLC22A12* ^10^, have major effects on the progression of gout/hyperuricemia. Certain environmental factors appear also to be risk factors for gout/hyperuricemia, of which alcohol consumption is one of the best known. Ethanol is oxidized to acetaldehyde by alcohol dehydrogenase (ADH), and acetaldehyde is further metabolized to acetate by aldehyde dehydrogenase (ALDH)^11^. These processes crucially depend on ADH1B and ALDH2, respectively (Fig. 1). We recently performed a genome-wide association study (GWAS) of gout^4^ followed by a fine-mapping study^12^ that identified rs671 (Glu504Lys) of *ALDH2* as a gout locus^12^. On the other hand, to our knowledge, the association between gout and common variants of *ADH1B* has not hitherto been reported. Additionally, there are no association analysis reports between gout and common variants of *ADH1B* and *ALDH2* that include adjustment for alcohol consumption. We therefore performed an association analysis between gout and a common dysfunctional variant of *ADH1B*, rs1229984 (His48Arg). We further investigated the effects of alcohol consumption on the association between gout and common variants of *ADH1B* and *ALDH2*.

## Results

### Association analysis between gout and common variants of *ADH1B* and *ALDH2*

We performed genotyping of rs1229984 (His48Arg) of *ADH1B* using 1,048 clinically defined gout cases and 1,334 controls of Japanese male (Table 1). The results are shown in Table 2 and Supplementary Table S1. The call rate for rs1229984 was 98.4%: this variant in the control group was in Hardy-Weinberg equilibrium (*P* > 0.05). The common dysfunctional variant of *ADH1B*, rs1229984, showed a significant association with gout for each allele model (*P* = 0.037; odds ratio [OR] = 1.16; 95% confidence interval [CI]: 1.01–1.34; Table 2). As shown in Supplementary Table S1, A/G (His/Arg) and A/A (His/His) genotypes significantly increase the risk of gout (*P* = 0.020 and 7.3 × 10^−3^; OR = 1.69 and 1.80, respectively) as compared with the G/G (Arg/Arg) genotype; however, there is no significant difference in effect sizes on gout between A/G (His/Arg) and A/A (His/His) genotypes (*P* = 0.51), and the OR is close to 1.00 (OR = 0.94; 95% CI: 0.79–1.12). Based on these results and enzyme activity^13–15^, we also performed an association analysis in the “His carrier (His/His or His/Arg)” (high tolerance for alcohol) vs. “non-His carrier (Arg/Arg)” (low tolerance for alcohol) model. Our results showed that the presence of “His carrier” significantly increased the risk of gout (*P* = 4.3 × 10^−4^; OR = 1.76; 95% CI: 1.15–2.69; Table 3). In addition, although not significant, the “A” allele (His) of *ADH1B* tended to increase alcohol consumption in controls (*P* = 0.14; 149.0 g/week for G/G, 155.7 g/week for A/G and 194.5 g/week for A/A; Supplementary Table S2). We further performed a logistic regression analysis that included alcohol consumption in the model using the classification of drinker or non-drinker. rs1229984 of *ADH1B* showed a significant association with gout, even after adjustment for alcohol consumption (*P* = 6.1 × 10^−3^; OR = 1.83; 95% CI: 1.19–2.81; Supplementary Table S3). Moreover, this association was also significant in the analysis conducted in drinkers only (*P* = 0.013) and the OR was similar to that in the analysis conducted in all participants (OR = 1.77; 95% CI: 1.13–2.78; Table 3); however, it proved not significant in the analysis conducted in non-drinkers only (*P* = 0.24; OR = 2.48; 95% CI: 0.55–11.2; Table 3), although the direction of OR is the same as that seen in the analysis conducted in all participants and in drinkers only; however, the sample size of non-drinkers is relatively small.

**Table 1:** Clinical characteristics of participants.

	Gout cases	Controls
Number	1,048	1,334
Age (year)	44.9 ± 11.4	52.4 ± 8.6
Body-mass index (kg/m^2^)	25.0 ± 3.5	23.2 ± 2.7
Alcohol drinker^*^	930 (88.7%)	1,011 (75.8%)

Plus-minus values are means ± SD.

^*^Participants who consumed alcohol at least once a month were classified as drinkers.

**Table 2:** Association analysis between gout and two common variants of *ADH1B* and *ALDH2*.

Gene	SNP	Genotype	Amino acid	Gout cases	Controls	*P* value^*^	OR (95%CI)^†^
*ADH1B*	rs1229984	G/G	Arg/Arg	32	71	0.037	1.16 (1.01–1.34)
		A/G	His/Arg	348	456		
		A/A	His/His	643	793		
*ALDH2*	rs671^‡^	A/A	Lys/Lys	48	108	1.7 × 10^−18^	1.88 (1.63–2.16)
		A/G	Lys/Glu	270	556		
		G/G	Glu/Glu	729	670		

Abbreviations: SNP = single nucleotide polymorphism; OR = odds ratio; CI = confidence interval; His = histidine; Arg = arginine; Glu = glutamic acid; Lys = lysine.

^*^The *P* values were calculated using logistic regression analysis.

^†^The ORs were calculated per allele model. For rs1229984 (His48Arg), “A” is the risk allele. For rs671 (Glu504Lys), “G” is the risk allele.

^‡^The genotyping results of rs671 are obtained from our previous report^12^.

**Table 3:** Effect of *ADH1B* and *ALDH2* genotypes and alcohol consumption on gout susceptibility.

Gene	SNP	Genotype	Amino acid	All participants				Only drinkers^*^				Only non-drinkers^*^			
				Gout cases	Controls	*P* value^†^	OR (95%CI)	Gout cases	Controls	*P* value^†^	OR (95%CI)	Gout cases	Controls	*P* value^†^	OR (95%CI)
*ADH1B*	rs1229984	A/A or A/G	His carrier	991	1,249	4.3 × 10^−4^	1.76^‡^ (1.15–2.69)	877	941	0.013	1.77^‡^ (1.13–2.78)	114	299	0.24	2.48^‡^ (0.55–11.2)
		G/G	non-His carrier	32	71	—	Reference	30	57	—	Reference	2	13	—	Reference
*ALDH2*	rs671	G/G	non-Lys carrier	729	670	2.9 × 10^−21^	2.27^§^ (1.92–2.69)	703	625	7.2 × 10^−11^	1.92^§^ (1.58–2.34)	26	40	0.021	1.93^§^ (1.12–3.33)
		A/A or A/G	Lys carrier	318	664	—	Reference	226	386	—	Reference	92	273	—	Reference

Abbreviations: OR = odds ratio; CI = confidence interval; His = histidine; Lys = lysine.

^*^Participants who consumed alcohol less than once a month were classified as non-drinkers.

^†^The *P* values were calculated using logistic regression analysis.

^‡^For rs1229984 (His48Arg), A/A (His/His) or A/G (His/Arg) genotype (His carrier, high tolerance for alcohol) is a risk, so the “His carrier” vs. “non-His carrier” model was used for the analysis of rs1229984.

^§^For rs671 (Glu504Lys), G/G (Glu/Glu) genotype (non-Lys carrier, high tolerance for alcohol) is a risk, so the “non-Lys carrier” vs. “Lys carrier” model was used for rs671.

We have previously demonstrated an association between rs671 (Glu504Lys) of *ALDH2* and gout^12^ as also shown in Table 2. In addition, as shown in Supplementary Table S1, A/G (Lys/Glu) and A/A (Lys/Lys) genotypes of *ALDH2* significantly decrease the risk of gout (*P* = 3.8 × 10^−19^ and 4.8 × 10^−7^; OR = 0.45 and 0.41, respectively) as compared with the G/G (Glu/Glu) genotype; however, there is no significant difference in effect sizes on gout between A/G (Lys/Glu) and A/A (Lys/Lys) genotypes (*P* = 0.71), and the OR is close to 1.00 (OR = 1.09; 95% CI: 0.75–1.62). Based on these results and enzyme activity^16^, the “non-Lys carrier (Glu/Glu)” (high tolerance for alcohol) vs. “Lys carrier (Lys/Glu or Lys/Lys)” (low tolerance for alcohol) model was used for the following analysis. We also performed a multivariate logistic regression analysis that included alcohol consumption in the model because *ALDH2* genotypes were significantly associated with the proportion of non-drinkers (*P* = 2.5 × 10^−83^; 93.5% for A/A, 32.2% for A/G and 6.3% for G/G: Supplementary Table S2) and alcohol consumption in controls (*P* = 2.0 × 10^−51^; 0.68 g/week for A/A, 91.2 g/week for A/G and 231.0 g/week for G/G: Supplementary Table S2). The association between gout and rs671 of *ALDH2* remained significant even after adjustment for alcohol consumption (*P* = 4.3 × 10^−12^; OR = 1.92; 95% CI: 1.60–2.31: Supplementary Table S3). Contrary to the result for *ADH1B*, this association was still significant in the analysis conducted in both non-drinkers only and drinkers only, and the direction of OR and the effect size were similar to those obtained in the analysis conducted in all participants (*P* = 0.021 and 7.2 × 10^−11^; OR = 1.93 and 1.92; 95% CI: 1.12–3.33 and 1.58–2.34, respectively: Table 3).

### Gout risk due to combination of the *ADH1B* and *ALDH2* genotypes

Next, we investigated the combined effects on gout of the common variants of *ADH1B* (rs1229984) and *ALDH2* (rs671). Based on enzyme activity^13–15^, the “His carrier (His+)” vs. “non-His carrier (His−)” model was selected for the association analysis between gout and rs1229984 (His48Arg) of *ADH1B*. Regarding the association analysis between gout and rs671 (Glu504Lys) of *ALDH2*, we adopted the “non-Lys carrier (Lys−)” vs. “Lys carrier (Lys+)” model as described in our previous paper^12^. Individuals whose combination of rs1229984 and rs671 is “His−/Lys+”, “His−/Lys−” or “His+/Lys+” were subject to a significantly lower risk of gout (*P* = 3.0 × 10^−3^, 2.9 × 10^−3^ and 8.7 × 10^−22^, respectively) than the other group (“His +/Lys−”), as shown in Table 4. Furthermore, although the 95% CIs overlap each other, the OR of “His−/Lys+” (OR = 0.36; 95% CI: 0.18–0.71) is lower than those of “His−/Lys−” and “His+/Lys+” (OR = 0.44 and 0.42; 95% CI: 0.25–0.75 and 0.36–0.51, respectively).

**Table 4:** Gout risk due to combination of *ADH1B* and *ALDH2* genotypes.

rs1229984^*^ (*ADH1B*)	rs671^*^ (*ALDH2*)	Gout cases	Controls	*P* value^†^	OR (95% CI)
His−	Lys+	12	30	3.0 × 10^−3^	0.36 (0.18–0.71)
His−	Lys−	20	41	2.9 × 10^−3^	0.44 (0.25–0.75)
His+	Lys+	297	628	8.7 × 10^−22^	0.42 (0.36–0.51)
His+	Lys−	693	621	—	Reference

Abbreviations: His = histidine; Lys = lysine; OR = odds ratio; CI = confidence interval.

^*^In the analysis of rs1229984 (His48Arg), “His+” and “His−“ mean His carrier (His/His or His/Arg) and non-His carrier (Arg/Arg), respectively. In the analysis of rs671 (Glu504Lys), “Lys+” and “Lys−” mean Lys carrier (Lys/Lys or Lys/Glu) and non-Lys carrier (Glu/Glu), respectively. We investigated the combined effects of rs1229984 and rs671 on gout as compared with “His +/Lys−”.

^†^The *P* value was calculated using logistic regression analysis.

## Discussion

ADH1B and ALDH2 are crucial enzymes for alcohol metabolism, and it is already established that individual differences in these two enzymes’ activities are caused by common variants^13^. The functionally important variants for *ADH1B* are rs1229984 (His48Arg) and rs2066702 (Arg370Cys)^17–19^. The allele frequencies of rs1229984 and rs2066702 of *ADH1B* differ among populations, according to the results of a previous paper^13^ and ISGR’s 1000 Genomes Phase 3^20^. rs1229984 is polymorphic in Europeans and East Asians, including Japanese, while it is monomorphic in Africans. On the other hand, rs2066702 is monomorphic in Europeans and East Asians but polymorphic in Africans. In this study, therefore, we genotyped rs1229984 with Japanese participants. Because the A/A (His/His) or A/G (His/Arg) genotype of rs1229984 has been reported to produce 40-fold faster ethanol oxidation than the G/G (Arg/Arg) genotype^13–15^, in the present study, we investigated not only the genotype model but also the “His carrier” vs. “non-His carrier” model for the analysis of rs1229984. Regarding the analysis of *ALDH2*, rs671 (Glu504Lys) is a noted functional variant^16, 21^. The Lys allele of rs671 is common in East Asians, but quite rare in Europeans and Africans^20, 22^. Individuals with heterozygotes (Lys/Glu) of rs671 have only 6.25% of the enzyme activity of those with normal ALDH2 (Glu/Glu), and those with homozygotes (Lys/Lys) show almost no activity^16^. We therefore adopted the “non-Lys carrier” vs. “Lys carrier” model for rs671 in the present study.

No reports on the association between gout and common variants of *ADH1B* have been published, although Yokoyama *et al*. recently reported that a common dysfunctional variant of *ADH1B*, rs1229984, is associated with serum uric acid (SUA) levels in male Japanese alcoholics^23^. In this study, for the first time, we revealed a significant association between a common dysfunctional variant of *ADH1B* (rs1229984) and gout (Table 2 and Supplementary Table S1).

We previously reported the association between gout and rs671 of *ALDH2* ^12^. Other Japanese^24^ and Chinese^25^ studies have also indicated this association. However, in these studies^12, 24, 25^, alcohol consumption was not taken into consideration, even though rs671 is associated with alcohol consumption (Supplementary Table S2). Thus, we first investigated the association between gout and rs671 of *ALDH2* including alcohol consumption in the model. The common dysfunctional variant of *ALDH2*, rs671, also showed a significant association with gout, even after adjustment for alcohol consumption (Supplementary Table S3) and even in non-drinkers or in drinkers (Table 3). On the other hand, although the association between gout and rs1229984 of *ADH1B* was still significant even after adjustment for alcohol consumption (Supplementary Table S3) and in drinkers (Table 3), this association was not significant in non-drinkers (Table 3). Because the sample size of non-drinkers was relatively small, further studies are necessary to clarify the effects of alcohol consumption on the association between gout and common variants of *ADH1B* and *ALDH2*.

It appears that alcohol intake elevates SUA level by increasing urate production^26, 27^ and decreasing renal urate excretion^28^. Ethanol is oxidized to acetate mainly by ADH1B and ALDH2 (Fig. 1). When acetate is further metabolized to acetyl-coenzyme A, adenosine triphosphate (ATP) hydrolyzes to adenosine monophosphate (AMP), which is ultimately metabolized to urate. Thus, alcohol consumption could increase urate by enhancing hydrolysis from ATP to AMP^27^. Furthermore, the “His+/Lys−” genotype combination causes faster ethanol and acetaldehyde elimination and may accelerate the increase in ATP degradation, which further elevates SUA^23^. This may be one of the reasons why “His+/Lys−” tends to have a stronger effect on gout than other genotype combinations, in spite of the 95% CIs overlapping each other (Table 4). It is also well known that alcohol consumption can increase lactate^29^ which is exchanged for urate via urate transporter 1 (URAT1/SLC22A12) in the human kidney^30^. Therefore, alcohol consumption could also increase the SUA level by enhancing the renal urate reabsorption via URAT1. Taking into consideration the factors mentioned above, alcohol consumption could increase the risk of gout susceptibility resulting from hyperuricemia. ADH1B and ALDH2 enzyme activities, which depend on the common variants, affect alcohol consumption behavior, and the genotyping of *ADH1B* and *ALDH2* variants can be a surrogate for alcohol consumption in the estimation of risks for several diseases, including esophageal cancer, which were demonstrated by Mendelian randomization approaches^31, 32^. Thus, we initially assumed that the associations between gout and common variants of *ADH1B* and *ALDH2* would be accounted for by alcohol consumption. Contrary to this expectation, these associations were still significant even after adjustment for alcohol consumption (Supplementary Table S3), which indicates that common variants of *ADH1B* and *ALDH2* can be associated with gout susceptibility through not only alcohol consumption but also other factors and/or mechanisms. However, the association of *ADH1B* was not significant in non-drinkers (Table 3). This study had several limitations in that we were able to use only the frequency data, not the quantity data, on alcohol consumption by gout cases. Similarly, the adjustment for alcohol consumption might not be sufficient because these alcohol-drinking data were self-reported, and it is difficult to obtain data on lifetime alcohol consumption. A further problem is that adjustment of the association between these genetic variants and gout for alcohol consumption could also lead to collider bias. It is similar that the adjustment for cigarettes smoked per day does not entirely mediate the relationship between genetic variants and lung cancer: this is most likely due to the fact that daily cigarette consumption does not accurately capture total tobacco exposure^33^. Therefore, from the point of view of alcohol consumption, further studies are necessary to be able to elucidate the association between gout and common variants of *ADH1B* and *ALDH2*.

In summary, our data show that common variants of *ADH1B* (rs1229984) and *ALDH2* (rs671) are independently associated with gout, which indicates that the genotyping of rs1229984 and rs671 can be useful for the evaluation of gout risk.

## Methods

### Study participants

This study was approved by the institutions’ Ethical Committees (National Defense Medical College and Nagoya University). All procedures were performed in accordance with the Declaration of Helsinki, with written informed consent obtained from each subject. In this study, all the participants were Japanese males: the frequency of Japanese female gout patients is extremely low, at about only 1% of the entire population of gout patients that we analyzed. The gout cases comprised 1,048 patients assigned from Japanese male outpatients at the gout clinics of Kyoto Industrial Health Association (Kyoto, Japan) or Ryougoku East Gate Clinic (Tokyo, Japan). All patients were clinically diagnosed with primary gout according to the criteria established by the American College of Rheumatology^34^. Patients with inherited metabolic disorders, including Lesch–Nyhan syndrome, were excluded. For the control group, 1,334 Japanese males with SUA levels of ≤7.0 mg/dl and without a history of gout were recruited from the participants in the Shizuoka area in the Japan Multi-Institutional Collaborative Cohort Study (J-MICC Study)^35, 36^. Participants who consumed alcohol at least once a month were classified as drinkers. In the controls, the information on alcohol consumption was collected at the point of recruitment into the study. Meanwhile, in the gout cases, we used information on alcohol consumption at the point of gout onset. There is detailed information on alcohol consumption for the controls: we show and analyze the amount of alcohol consumption data for each genotype (Supplementary Table S2). On the other hand, the information on alcohol consumption in gout cases was limited to whether the subject is a drinker or non-drinker. Thus, in this study, the adjustment for alcohol consumption was performed using the classification of drinker or non-drinker. The details on the participants in this study are shown in Table 1.

### Genetic analysis

Genomic DNA was extracted from whole peripheral blood cells^37^. Genotyping of rs1229984 of *ADH1B* was performed using the TaqMan method (Thermo Fisher Scientific, Waltham, MA, USA) employing a LightCycler 480 (Roche Diagnostics, Mannheim, Germany)^37^ with minor modifications. The custom TaqMan assay probe was designed as follows: VIC- CTGTAGGAATCTGTCACACAG and FAM- TGTAGGAATCTGTCGCACAG. Genotyping data on rs671 of *ALDH2* was obtained from our previous study^12^.

### Statistical analyses

R-3.1.1 (http://www.r-project.org/) software was used for all calculations in the statistical analysis^38^. The association analyses were examined using Fisher’s exact test, Cochran-Armitage test, linear regression analysis and logistic regression analysis. All *P* values were two-tailed and *P* values of <0.05 were regarded as statistically significant.

## Electronic supplementary material

Supplementary Information
