# Supplementary material for: Independent effects of ADH1B and ALDH2 common dysfunctional variants on gout risk
Source: Sci Rep. 2017 May 31;7:2500. doi: 10.1038/s41598-017-02528-z (PMC5451470; doi:10.1038/s41598-017-02528-z)
Supplement: Supplementary file 1 — Supplementary Information [file 41598_2017_2528_MOESM1_ESM.doc]

SUPPLEMENTARY INFORMATION

**Independent effects of *ADH1B* and *ALDH2* common dysfunctional variants on gout risk**

Masayuki Sakiyama*, Hirotaka Matsuo*, Airi Akashi, Seiko Shimizu,

Toshihide Higashino, Makoto Kawaguchi, Akiyoshi Nakayama, Mariko Naito,

Sayo Kawai, Hiroshi Nakashima, Yutaka Sakurai, Kimiyoshi Ichida, Toru Shimizu, Hiroshi Ooyama and Nariyoshi Shinomiya

* These authors contributed equally to this work.

Corresponding Author: Hirotaka Matsuo (hmatsuo@ndmc.ac.jp)

**Supplementary Table S1** | Effect sizes on gout for each genotype of two common variants of *ADH1B* and *ALDH2*

**Supplementary Table S2** | Relation between alcohol consumption and genotypes of *ADH1B* and *ALDH2* in controls

**Supplementary Table S3** | Effect of *ADH1B* and *ALDH2* genotypes and alcohol consumption on gout susceptibility

**Supplementary Table S1. Effect sizes on gout for each genotype of two common variants of *ADH1B* and *ALDH2***

| Gene | SNP | Genotype | Amino acid | Gout cases | Controls | *P* value^*^ | OR (95%CI) ^†^ | *P* value^*^ | OR (95%CI) ^†^ |
| --- | --- | --- | --- | --- | --- | --- | --- | --- | --- |
| *ADH1B* | rs1229984 | G/G | Arg/Arg | 32 | 71 | 7.3 × 10^–3^ | 0.56 (0.35-0.87) | – | Reference |
|  |  | A/G | His/Arg | 348 | 456 | 0.51 | 0.94 (0.79-1.12) | 0.020 | 1.69 (1.07-2.72) |
|  |  | A/A | His/His | 643 | 793 | – | Reference | 7.3 × 10^–3^ | 1.80 (1.15-2.86) |
| *ALDH2* | rs671 | A/A | Lys/Lys | 48 | 108 | 4.8 × 10^–7^ | 0.41 (0.28-0.59) | – | Reference |
|  |  | A/G | Lys/Glu | 270 | 556 | 3.8 × 10^–19^ | 0.45 (0.37-0.54) | 0.71 | 1.09 (0.75-1.62) |
|  |  | G/G | Glu/Glu | 729 | 670 | – | Reference | 4.8 × 10^–7^ | 2.45 (1.70-3.57) |

Abbreviations: SNP = single nucleotide polymorphism; OR = odds ratio; CI = confidence interval; His = histidine; Arg = arginine; Glu = glutamic acid; Lys = lysine.

^*^The*P* values were calculated using Fisher’s exact test.

^†^The ORs were calculated per genotype. For rs1229984 (His48Arg), "A" is the risk allele. For rs671 (Glu504Lys), "G" is the risk allele.

**Supplementary Table S2. Relation between alcohol consumption and genotypes of *ADH1B* and *ALDH2* in controls**

| Gene | SNP | Genotype | Amino acid | Number | Non-drinkers^*^ (%) | *P* value^†^ | Alcohol consumption^‡^ (g/week of pure alcohol) | *P* value^§^ |
| --- | --- | --- | --- | --- | --- | --- | --- | --- |
| *ADH1B* | rs1229984 | G/G | Arg/Arg | 66 | 13 (19.7%) |  | 149.0 ± 199.8 |  |
|  |  | A/G | His/Arg | 439 | 104 (23.7%) |  | 155.7 ± 195.9 |  |
|  |  | A/A | His/His | 763 | 195 (25.6%) | 0.25 | 194.5 ± 219.0 | 0.14 |
| *ALDH2* | rs671 | A/A | Lys/Lys | 108 | 101 (93.5%) |  | 0.68 ± 3.3 |  |
|  |  | A/G | Lys/Glu | 534 | 172 (32.2%) |  | 91.2 ± 131.3 |  |
|  |  | G/G | Glu/Glu | 639 | 40 (6.3%) | 2.5 × 10^–83^ | 231.0 ± 228.3 | 2.0 × 10^–51^ |

Abbreviations: His = histidine; Arg = arginine; Glu = glutamic acid; Lys = lysine.

^*^Participants who consumed alcohol less than once a month were classified as non-drinkers.

^†^The *P* values were calculated using Cochran-Armitage test.

^‡^Alcohol consumption was calculated from patricipants’ written questionnaires as same as our previous report.^1^

^§^The *P* values were calculated using linear regression analysis.

**Reference**

1. Nakayama, A. *et al.* Common dysfunctional variants of ABCG2 have stronger impact on hyperuricemia progression than typical environmental risk factors. *Sci. Rep.* **4**, 5227 (2014).

**Supplementary Table S3. Effect of *ADH1B* and *ALDH2* genotypes and alcohol consumption on gout susceptibility**

| Gene | SNP | Genotype | Amino acid | Gout cases | Controls | *P* value^*^ | OR (95%CI) | Adjusted *P* value^†^ | Adjusted  OR (95%CI)^†^ | |
| --- | --- | --- | --- | --- | --- | --- | --- | --- | --- | --- |
| *ADH1B* | rs1229984 | A/A or A/G | His carrier | 991 | 1249 | 4.3 × 10^–4^ | 1.76 (1.15 - 2.69)^‡^ | 6.1 × 10^–3^ | | 1.83 (1.19 - 2.81) |
|  |  | G/G | non-His carrier | 32 | 71 | – | Reference | – | | Reference |
| *ALDH2* | rs671 | G/G | non-Lys carrier | 729 | 670 | 2.9 × 10^–21^ | 2.27 (1.92 - 2.69)^§^ | 4.3 × 10^–12^ | | 1.92 (1.60 - 2.31) |
|  |  | A/A or A/G | Lys carrier | 318 | 664 | – | Reference | – | | Reference |

Abbreviations: OR = odds ratio; CI = confidence interval; His = histidine; Lys = lysine.

^*^The*P* value was calculated using logistic regression analysis.

^†^The *P* value and OR were adjusted for alcohol consumption.

^‡^For rs1229984 (His48Arg), A/A (His/His) or A/G (His/Arg) genotype (His carrier, high tolerance for alcohol) is risk, then the “His carrier” vs. “non-His carrier” model was used for the analysis of rs1229984.

^§^For rs671 (Glu504Lys), G/G (Glu/Glu) genotype (non-Lys carrier, high tolerance for alcohol) is risk, then “non-Lys carrier” vs. “Lys carrier” model was used for rs671
